# Supplementary material for: Oxalis tetraphylla (Class: Magnoliopsidae) Possess Flavonoid Phytoconstituents with Nematocidal Activity against Haemonchus contortus
Source: Pathogens. 2022 Sep 8;11(9):1024. doi: 10.3390/pathogens11091024 (PMC9503173; doi:10.3390/pathogens11091024)
Supplement: Supplementary file 1 [file pathogens-11-01024-s001.zip › pathogens-1882063-supplementary.pdf]

A

B

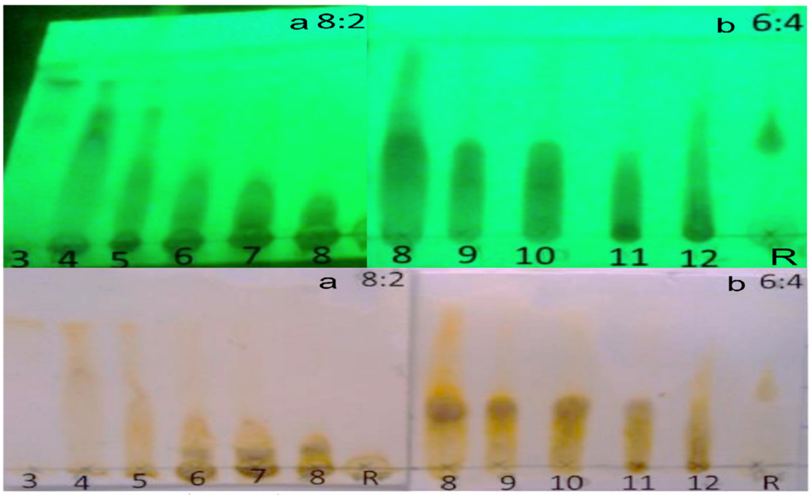

Figure S1. **A**) TLC plate from *Oxalis tetraphylla* aqueous phase (normal phase) visualized with UV light; numbers correspond to eluted fractions collected from two DCM:MeOH systems: a) system 8:2, fractions 3 to 7 and b) system 6:4, fractions 8 to 12. R=Routine (standard). **B**) Same plats, developed with flavonoid reagent showing bands associated with the presence of flavonoids at two DCM:MeOH systems, were visualized: a) system 8.2, fractions 3 to 8 and b) system 6:4, fractions 8 to 12.
